# Author Correction: The gut microbiota in infants of obese mothers increases inflammation and susceptibility to NAFLD

**DOI:** 10.1038/s41467-019-10943-1

**Published:** 2019-07-01

**Authors:** Taylor K. Soderborg, Sarah E. Clark, Christopher E. Mulligan, Rachel C. Janssen, Lyndsey Babcock, Diana Ir, Bridget Young, Nancy F. Krebs, Dominick J. Lemas, Linda K. Johnson, Tiffany Weir, Laurel L. Lenz, Daniel N. Frank, Teri L. Hernandez, Kristine A. Kuhn, Angelo D’Alessandro, Linda A. Barbour, Karim C. El Kasmi, Jacob E. Friedman

**Affiliations:** 1 Department of Pediatrics, Section of Neonatology, University of Colorado Anschutz Medical Campus, Aurora, 80045 CO USA; 2 Department of Microbiology and Immunology, University of Colorado Anschutz Medical Campus, Aurora, 80045 CO USA; 3 Department of Medicine, Division of Infectious Disease, University of Colorado Anschutz Medical Campus, Aurora, 80045 CO USA; 4 Department of Pediatrics, Section of Nutrition, University of Colorado Anschutz Medical Campus, Aurora, 80045 CO USA; 5 Department of Pathology, University of Colorado Anschutz Medical Campus, Aurora, 80045 CO USA; 6 Department of Food Science and Human Nutrition, Colorado State University, Fort Collins, 80523 CO USA; 7 Department of Medicine, Division of Endocrinology, Metabolism & Diabetes, University of Colorado Anschutz Medical Campus, Aurora, 80045 CO USA; 8 College of Nursing, University of Colorado Anschutz Medical Campus, Aurora, 80045 CO USA; 9 Department of Medicine, Division of Rheumatology, University of Colorado Anschutz Medical Campus, Aurora, 80045 CO USA; 10 Department of Biochemistry and Molecular Genetics, University of Colorado Anschutz Medical Campus, Aurora, 80045 CO USA; 11 Department of Obstetrics and Gynecology, Division of Maternal Fetal Medicine, University of Colorado Anschutz Medical Campus, Aurora, 80045 CO USA; 12 Department of Pediatrics, Section of Gastroenterology, Hepatology and Nutrition, University of Colorado Anschutz Medical Campus, Aurora, 80045 CO USA; 13 Department of Pediatrics; Allergy and Immunology, University of Rochester School of Medicine and Dentistry, Rochester, NY 14642 USA; 14 Department of Health Outcomes and Biomedical Informatics, University of Florida, Gainsville, FL 32610 USA

**Keywords:** Microbiome, Obesity, Non-alcoholic fatty liver disease, Risk factors

Correction to: *Nature Communications* 10.1038/s41467-018-06929-0; published online 26 October 2018

The original version of this Article omitted from the author list the 7th author Bridget Young, who is from the ‘Department of Pediatrics, Section of Nutrition, University of Colorado Anschutz Medical Campus, Aurora 80045 CO, USA’ and ‘Present address: Department of Pediatrics; Allergy and Immunology, University of Rochester School of Medicine and Dentistry, Rochester, NY 14642, USA’, and the 8th author Nancy F. Kriebs, who is from the ‘Department of Pediatrics, Section of Nutrition, University of Colorado Anschutz Medical Campus, Aurora 80045 CO, USA’.

Consequently, ‘L.A.B., and N.F.K.’ was added to the fourth sentence of the Acknowledgements: ‘This study was supported by the American Diabetes Association/Glaxo Smith Kline Targeted Research Award (1-13-GSK-13, to J.E.F., L.A.B., and N.F.K.), […]’. Plus the following was added to the end of the Acknowledgements: ‘B.Y. and N.F.K. were supported by NIH/National Institute of Diabetes and Digestive and Kidney Diseases T32 DK007658 and NIH/Child Health and Development grant F32-0978068 (B.Y.).’

In addition, the following was added to the Author contributions: ‘B.Y. and N.F.K. helped to design the clinical study and participated in the sample collection during the infant visits.’

This has been corrected in both the PDF and HTML versions of the Article.

