# Supplementary material for: The gut microbiota in infants of obese mothers increases inflammation and susceptibility to NAFLD
Source: Nat Commun. 2018 Oct 26;9:4462. doi: 10.1038/s41467-018-06929-0 (PMC6203757; doi:10.1038/s41467-018-06929-0)
Supplement: Supplementary file 1 — Supplementary Information [file 41467_2018_6929_MOESM1_ESM.pdf]

Supplementary materials

The gut microbiota in infants of obese mothers increases inflammation and susceptibility to NAFLD

Soderborg et al.

Supplementary Table 1. Select maternal dietary macro- and micronutrients two-weeks postpartum

	Normal-weight	Obese	*P* value*
Energy (kcal)	2316.99 ± 267.02	2580.08 ± 187.65	0.451
Total fat (g)	91.29 ± 8.84	104.85 ± 12.05	0.399
Total carbohydrate (g)	286.50 ± 42.72	307.91 ± 18.48	0.662
Total protein (g)	93.58 ± 11.07	107.16 ± 9.31	0.384
Total dietary fiber (g)	23.24 ± 3.77	19.39 ± 1.87	0.408
Choline (mg)	351.66 ± 50.13	368.49 ± 51.29	0.822

Results are expressed as mean ± SEM.
**P* value assessed by Student's *t*-test.

Supplementary Table 2. Forward and reverse sequences of primers used for qPCR

Gene Symbol	Forward Primer	Reverse Primer
18S	TCCGATAACGAACGAGAC	CTAAGGGCATCACAGACC
Abcb11	GACTTTCCACAGTGGCGTCT	TCACTCAACAACCCTACAGATG
Cpt1a	GTCCCTCCAGCTGGCTTATC	CATGCGGCCAGTGGTGTCTA
Cyp7a1	TGATCCTCTGGGCATCTCAAGCAA	AGCTCTTGGCCAGCACTCTGTAAT
Cyp8b1	TTGCAAATGCTGCCTCAACC	TAACAGTCGCACACATGGCT
Gapdh	GTGAAGGTCGGTGTGAACGGATTT	TGGCAACAATCTCCACTTTGCCAC
Hnf4a	GGATATGGCCGACTACAGCG	AGATGGGGACGTGTCATTGC
Ifnb1	AACTCCACCAGCAGACAGTG	GGTACCTTTGCACCCTCCAG
Il10	GCTCTTGCACTACCAAAGCC	CTGCTGATCCTCATGCCAGT
Il1b	GCCTTGGGCCTCAAAGGAAAGAAT	ATTGCTTGGGATCCACACTCTCCA
Il6	TCCATCCAGTTGCCTTCTTG	TTTCTCATTTCCACGATTTCCC
Nr0b2	TCCTCTTCAACCCAGATGTGC	AGACTTCACACAGTGCCCAG
Nr1h4	TGGCTGAATGTATGTATACAGGTTT	CAGCGTGCTGCTTCACATTT
Ocln	TTGAACTGTGGATTGGCAGC	CAAGATAAGCGAACCTTGGCG
Ppara	TGCCCTGAACATCGAGTGTCGAAT	TCGTACACCAGCTTCAGCCGAATA
Pparg2	TTCGCTGATGCACTGCCTAT	GGAATGCGAGTGGTCTTCCA
Slc10a1	GTCCTCAAGGCAGGCATGAT	ATCAGGGAGGAGGTAGCCAG
Tjp1	GATGTTTATGCGGACGGTGG	CATTGCTGTGCTCTTAGCGG
Tnf	TAGCCCACGTCGTAGCAAAC	ACAAGGTACAACCCATCGGC
Xbp1s	CTGAGTCCGCAGCAGGTG	GGCAACAGTGTCAGAGTCCA

A

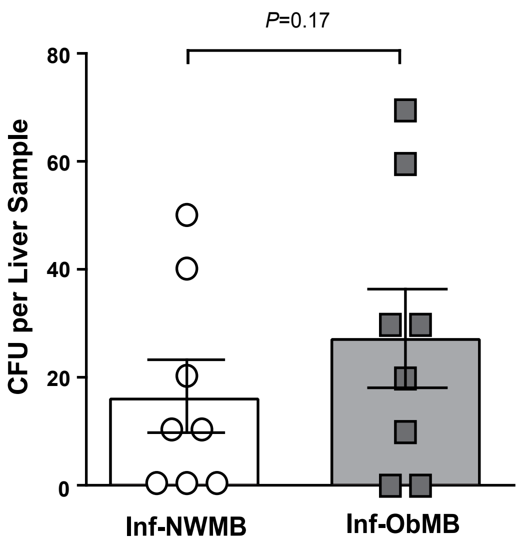

B

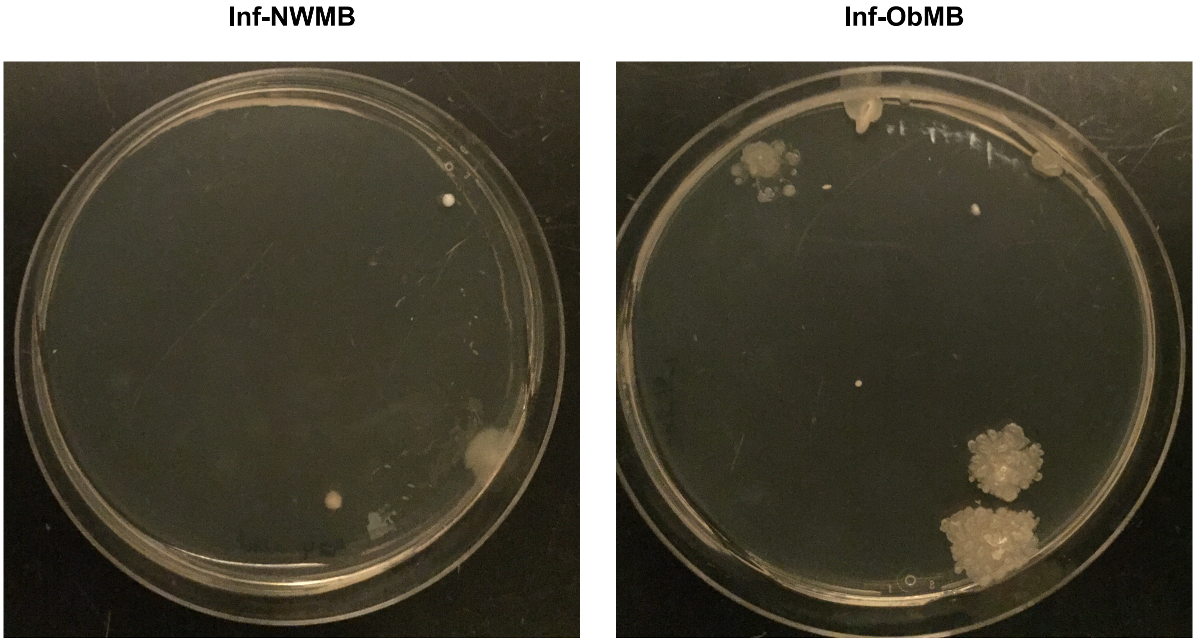

Supplementary Figure 1. Increased bacterial outgrowth from livers of Inf-ObMB mice.
A) Number of colony forming units (CFU) from liver homogenate on tryptic soy broth agar plates; n = 8 for both Inf-NWMB and Inf-ObMB. Data are presented as mean ± SEM. B) Fresh livers from Inf-NWMB and Inf-ObMB mice plated on tryptic soy broth agar plates and grown overnight at 37°C.

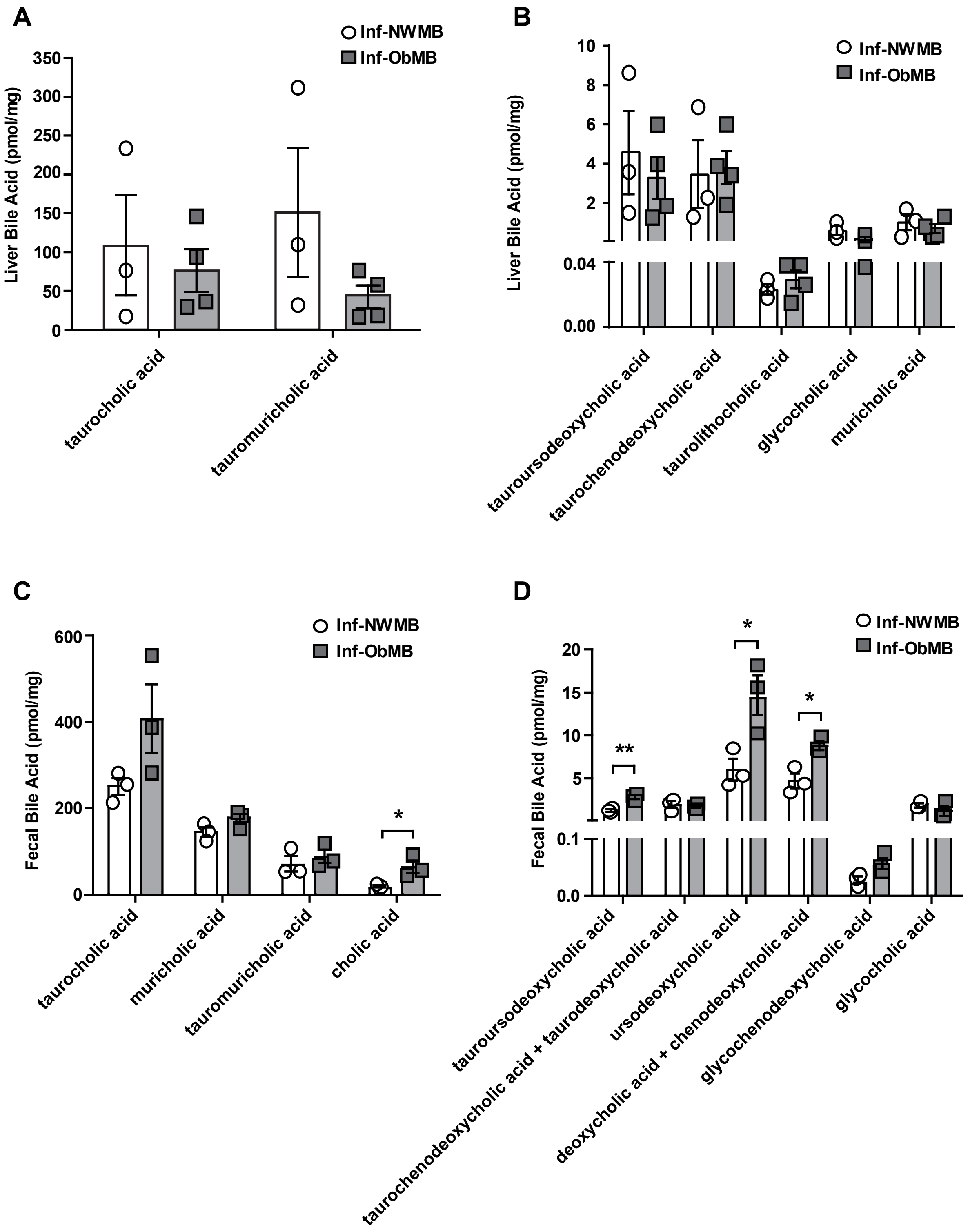

Supplementary Figure 2. Individual BA measurements in liver (A, B) and feces (C, D) in Inf-NWMB and Inf-ObMB mice. n = 3 for Inf-NWMB, n = 4 for Inf-ObMB for liver; n = 3 per group for feces. Data are presented as mean ± SEM. **P* <0.05 and ***P* <0.01 by Student's *t*-test.

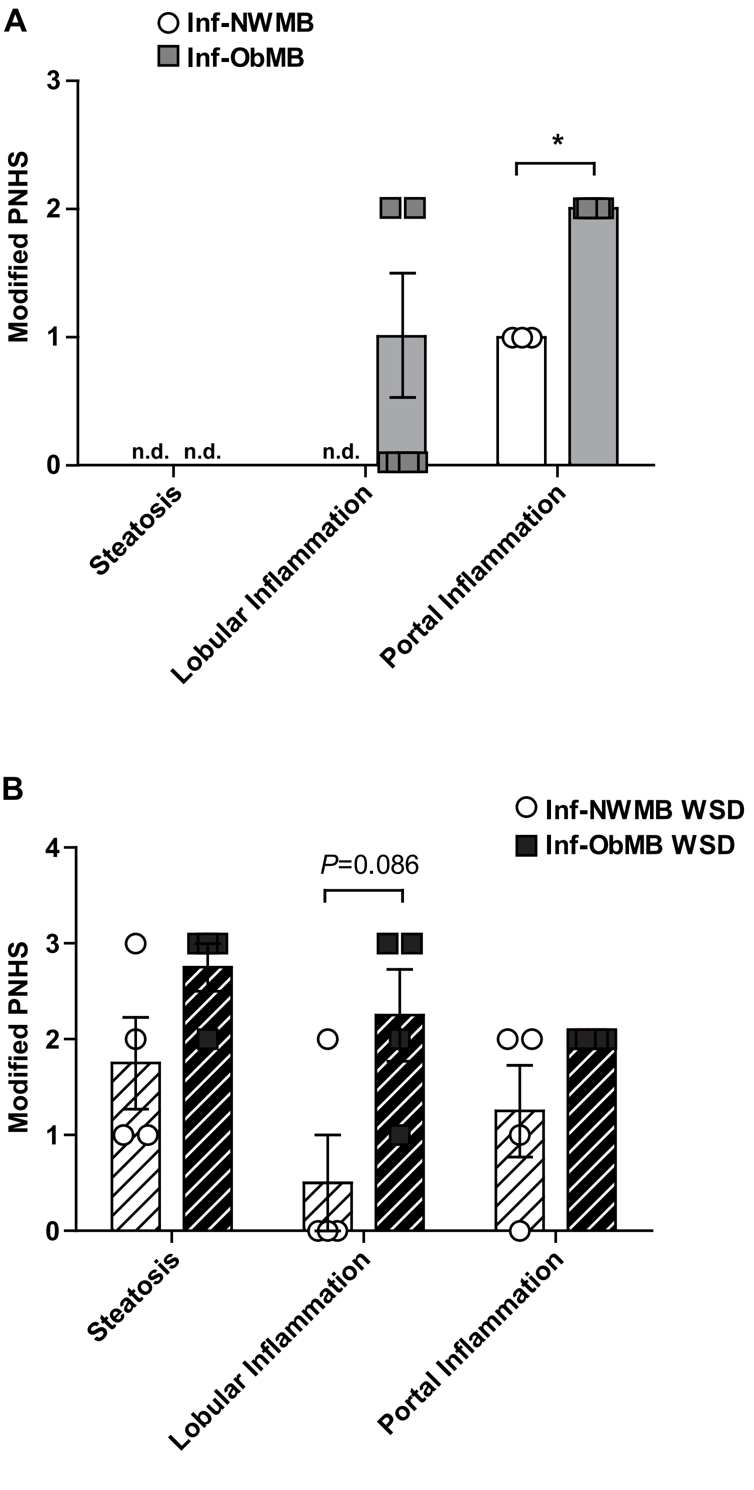

Supplementary Figure 3. Individual scores of the modified Pediatric NAFLD Histological Score (PNHS) for A) Inf-NWMB and Inf-ObMB mice and B) Inf-NWMB WSD and Inf-ObMB WSD mice. n = 3 for Inf-NWMB, n = 7 for Inf-ObMB, n = 4 for Inf-NWMB WSD and Inf-ObMB WSD. Data are presented as mean ± SEM. *P <0.05 by two-tailed Mann-Whitney U test.

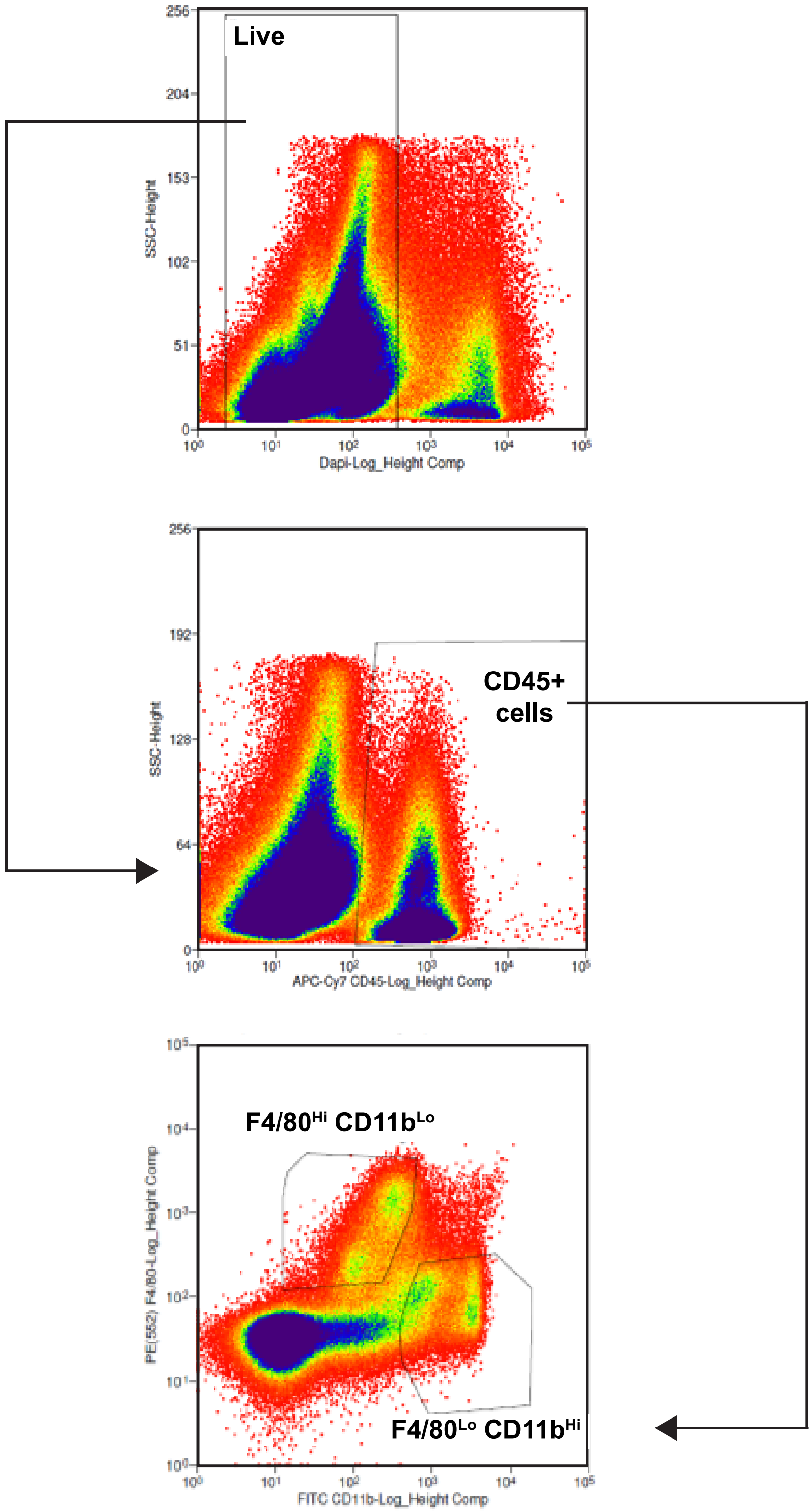

Supplementary Figure 4. Gating strategy for identifying resident and recruited liver macrophages.
