# Supplementary material for: The gut microbiota in infants of obese mothers increases inflammation and susceptibility to NAFLD
Source: Nat Commun. 2018 Oct 26;9:4462. doi: 10.1038/s41467-018-06929-0 (PMC6203757; doi:10.1038/s41467-018-06929-0)
Supplement: Supplementary file 3 — Reporting Summary [file 41467_2018_6929_MOESM3_ESM.pdf]

nature research

Corresponding author(s): Jacob Friedman

Reporting Summary

Nature Research wishes to improve the reproducibility of the work that we publish. This form provides structure for consistency and transparency in reporting. For further information on Nature Research policies, see Authors & Referees and the Editorial Policy Checklist.

Statistical parameters

When statistical analyses are reported, confirm that the following items are present in the relevant location (e.g. figure legend, table legend, main text, or Methods section).

n/a	Confirmed	
☐	☒	The exact sample size (n) for each experimental group/condition, given as a discrete number and unit of measurement
☐	☒	An indication of whether measurements were taken from distinct samples or whether the same sample was measured repeatedly
☐	☒	The statistical test(s) used AND whether they are one- or two-sided<br>*Only common tests should be described solely by name; describe more complex techniques in the Methods section.*
☒	☐	A description of all covariates tested
☒	☐	A description of any assumptions or corrections, such as tests of normality and adjustment for multiple comparisons
☐	☒	A full description of the statistics including central tendency (e.g. means) or other basic estimates (e.g. regression coefficient) AND variation (e.g. standard deviation) or associated estimates of uncertainty (e.g. confidence intervals)
☐	☒	For null hypothesis testing, the test statistic (e.g. F, t, r) with confidence intervals, effect sizes, degrees of freedom and P value noted<br>*Give P values as exact values whenever suitable.*
☒	☐	For Bayesian analysis, information on the choice of priors and Markov chain Monte Carlo settings
☒	☐	For hierarchical and complex designs, identification of the appropriate level for tests and full reporting of outcomes
☒	☐	Estimates of effect sizes (e.g. Cohen's d, Pearson's r), indicating how they were calculated
☐	☒	Clearly defined error bars<br>*State explicitly what error bars represent (e.g. SD, SE, CI)*

Our web collection on statistics for biologists may be useful.

Software and code

Policy information about availability of computer code

Data collection	N/A
Data analysis	GraphPad Prism 5 Software was used for data analysis, cellSens software was used for histological imaging, bile acid data was analyzed using MAVEN and flow cytometry was analyzed using FlowJo software.

For manuscripts utilizing custom algorithms or software that are central to the research but not yet described in published literature, software must be made available to editors/reviewers upon request. We strongly encourage code deposition in a community repository (e.g. GitHub). See the Nature Research guidelines for submitting code & software for further information.

Data

Policy information about availability of data

All manuscripts must include a data availability statement. This statement should provide the following information, where applicable:

- Accession codes, unique identifiers, or web links for publicly available datasets
- A list of figures that have associated raw data
- A description of any restrictions on data availability

16S rRNA sorted paired end sequence data were deposited in the NCBI Sequence Read Archive under accession number ‘PRJNA492901 [http://

www.ncbi.nlm.nih.gov/bioproject/492901]'. All other data supporting the findings of this study are available within the paper and its supplementary information files or are available from the corresponding author upon reasonable request.

Field-specific reporting

Please select the best fit for your research. If you are not sure, read the appropriate sections before making your selection.

☒ Life sciences ☐ Behavioural & social sciences ☐ Ecological, evolutionary & environmental sciences

For a reference copy of the document with all sections, see nature.com/authors/policies/ReportingSummary-flat.pdf

Life sciences study design

All studies must disclose on these points even when the disclosure is negative.

Sample size	Sample sizes were chosen based on availability of clinical samples and germ-free mouse availability.
Data exclusions	No data were excluded
Replication	We replicated this data by repeated mouse colonization with pooling of multiple unique stool samples from babies within each group. We achieved similar outcomes with each round of replication.
Randomization	Mice were colonized within littermate groups alternating between Inf-NWMB and Inf-ObMB.
Blinding	Histologist was blinded during the scoring of liver sections.

Reporting for specific materials, systems and methods

Materials & experimental systems

n/a	Involved in the study
☐	☒ Unique biological materials
☐	☒ Antibodies
☒	☐ Eukaryotic cell lines
☒	☐ Palaeontology
☐	☒ Animals and other organisms
☐	☒ Human research participants

Methods

n/a	Involved in the study
☒	☐ ChIP-seq
☐	☒ Flow cytometry
☒	☐ MRI-based neuroimaging

Unique biological materials

Policy information about availability of materials

Obtaining unique materials	Stool for colonization of germ-free mice was collected from human infants enrolled in a clinical study conducted by two of the authors on this manuscript. Therefore, we were limited in the availability of sample to the number of mother/infant dyads enrolled in the study. The materials are readily available to us as approved by the informed consent of the study participants.

Antibodies

Antibodies used	CD45-APC (clone 30-F11,Thermo #17-0451-82), F4/80-PE (clone BM8, Thermo #12-4801-82), CD11b-FITC (clone M1/70, Thermo #11-0112-41), CD11b-APC (clone M1/70, Thermo #17-0112-82), Anti-CD16/32 (2.4G2 hybridoma, ATCC #HB-197)
Validation	CD45-APC has been validated for reactivity to mouse for flow cytometry application by ThermoFisher Scientific. There are 58 publications cited using this antibody for flow cytometry. F4/80-PE has been validated for reactivity to mouse for flow cytometry application by ThermoFisher Scientific. There are 135 publications cited using this antibody for flow cytometry. CD11b-FITC has been validated for reactivity to mouse for flow cytometry application by ThermoFisher Scientific. There are 126 publications cited using this antibody for flow cytometry. CD11b-APC has been validated for reactivity to mouse for flow cytometry application by ThermoFisher Scientific. There are 152 publications cited using this antibody for flow cytometry. Anti-CD16/32 was generated from a 2.4G2 hybridoma obtained from ACTT and has been verified to have gene expression of and cellular production of immunoglobulin; monoclonal antibody; against the Fc gamma receptor (FcRII, CD32).

Animals and other organisms

Policy information about studies involving animals; ARRIVE guidelines recommended for reporting animal research

Laboratory animals	C57BL/6J male mice born and housed in a germ-free facility, aged 8 to 10 weeks old.
Wild animals	Study did not involve wild animals.
Field-collected samples	Study did not involve samples collected from the field.

Human research participants

Policy information about studies involving human research participants

Population characteristics	Women who were either normal weight or obese by pre-pregnancy BMI were eligible for this study. Only stool from infants who were born vaginally and were exclusively breastfed during the first 4 months was used in this study. Stool from male and female infants was used in this study.
Recruitment	Participants were recruited through a clinical study conducted by Drs. Barbour and Hernandez. The study is registered at clinicaltrials.gov (NCT01693406).

Flow Cytometry

Plots

Confirm that:

☒ The axis labels state the marker and fluorochrome used (e.g. CD4-FITC).

☒ The axis scales are clearly visible. Include numbers along axes only for bottom left plot of group (a 'group' is an analysis of identical markers).

☒ All plots are contour plots with outliers or pseudocolor plots.

☒ A numerical value for number of cells or percentage (with statistics) is provided.

Methodology

Sample preparation	Cells were isolated from collagenase digested livers. Cells were seperated using a 20% histodenz gradient and stained with antibodies to determine F4/80 and CD11b positivity.
Instrument	MoFlo XDP70 from Beckman Coulter
Software	FlowJo
Cell population abundance	Of the gated population, approximately 44% were resident and 0.8% were recruited for the Inf-NWMB and approximately 67% were resident and 5% were recruited for the Inf-ObMB group. Following a WSD, approximately 17% were resident and 5.3% were recruited for the Inf-NWMB and approximately 7.5% were resident and 15% were recruited for the Inf-ObMB group. Purity of samples was determined by using a fluorescence minus one (FMO) control paradigm on a set of control liver macrophages to set parameters for each antibody prior to running the samples.
Gating strategy	We first select live cells using Dapi+ gate. Then we identify CD45+ population from the Dapi+ cells. From the Dapi+CD45+ cells we identified resident liver macrophages by those that were high in F4/80 and low in CD11b and identified recruited liver macrophages as those that were low in F4/80 but high in CD11b.

☒ Tick this box to confirm that a figure exemplifying the gating strategy is provided in the Supplementary Information.
